# Implementation of Green Surgery Approach in Healthcare System and its Effect on Carbon Footprint Reduction in Operating Theatres

**DOI:** 10.21315/mjms-11-2024-884

**Published:** 2025-02-28

**Authors:** Thai Hau Koo, Xue Bin Leong, Yi Lin Lee, Mohd Hazeman Zakaria, Andee Dzulkarnaen Zakaria

**Affiliations:** 1 Department of Surgery, Hospital Pakar Universiti Sains Malaysia, Kelantan, Malaysia; 2 School of Medical Sciences, Universiti Sains Malaysia, Health Campus, Kelantan, Malaysia; 3 Radiology Department, Faculty of Medicine and Health Science, Universiti Putra Malaysia, Selangor, Malaysia; 4 Hospital Pakar Universiti Sains Malaysia, Kelantan, Malaysia

**Keywords:** Green surgery, carbon footprint, operating theatres, waste management, sustainability

## Abstract

Healthcare systems, particularly operating theatres (OTs), are among the leading causes of pollution due to energy-intensive procedures, anaesthetic gases, and single-use surgical instruments. This perspective review provides actionable, evidence-based recommendations that not only minimise the environmental impact but also offer quality patient care. A wide literature search of available studies on green surgery was conducted. Searches were conducted in databases, including PubMed, Science Direct, Scopus, ProQuest, and Web of Science, from January 2013 to the present. A review of global practices highlighted the effectiveness of green surgical initiatives. Some initiatives related to the health sector report reductions in carbon emissions by reusable surgical instrument implementation in hospitals of up to 97%, energy savings of up to 50% with modifications to heating, ventilation, and air-conditioning (HVAC) systems, and waste management programmes, including the recycling and reprocessing of single-use devices, reporting waste reductions from 40% to 66%. Key strategies include shifting to renewable energy sources by promoting reusable instruments, optimising HVAC systems, and promoting comprehensive staff training for sustainability. All these factors are important for decreasing the environmental burden without compromising operational efficiency. The integration of sustainable practices in Malaysian OTs can significantly reduce carbon emissions and waste generated by hospitals while maintaining patient safety. These measures support the national goals of achieving Sustainable Development Goals (SDGs) and advancing Universal Health Coverage (UHC). Aligning with global sustainability efforts, Malaysia’s health care system can reduce its carbon footprint.

## Introduction

Globally, healthcare systems have been identified as significant contributors to environmental pollution, with operating theatres (OTs) playing a notable role in hospitals’ carbon footprint (1). For instance, the US healthcare system generates 8%–10% of national emissions (2). Similarly, hospitals in the UK contribute 22.8 million tonnes of carbon dioxide (CO_2_) per year, representing 6% of the UK’s net CO_2_ emissions (3). Operating rooms (ORs) are among the most resource-intensive, generating three to six times more emissions per square foot than any other area in a hospital facility (2). OTs represent a significant amount of hospital waste and energy consumption; up to 60%–70% of hospital waste comes from OTs (4), mostly from disposable surgical instruments and other consumables (5). OTs are responsible for a substantial share of healthcare-related carbon emissions, primarily because of the high energy consumption required for HVAC systems, the use of anaesthetic gases, and waste generated from disposable surgical instruments and other consumables (6).

This growing concern regarding the environmental impact of healthcare, especially in OTs, has led to the integration toward integrating environmental sustainability into the clinical practice guidelines (CPGs) (7). In Malaysia, where the healthcare system is rapidly developing, there is an immediate need to address the carbon footprint of OTs by proposing sustainable practices. Energy-efficient designs, promotion of reusable surgical instruments, and better waste management strategies are just a few approaches considered to minimise the environmental impact of healthcare facilities (1).

Despite advancements in medical technology and healthcare delivery, OT practices for sustainability have not kept pace with the need for a reduction in the ecological impact. There is a clear gap in the current CPGs of Malaysia regarding environmentally sustainable health care practices. This study aimed to identify and evaluate current practices in OTs that contribute to carbon emissions, propose evidence-based recommendations for reducing the carbon footprint, and explore how Malaysia’s CPG can incorporate these practices to promote sustainability (1).

## Materials and Methods

### Research Design

The study design was a guideline for articles published on green surgery in the context of OTs using the Preferred Reporting Items for Systematic Review and Meta-Analysis (PRISMA) 2020 guidelines.

### Eligibility Criteria

Eligible articles included non-interventional and interventional studies based on the following criteria: i) “The article must consider green surgery practices or innovations”; ii) “study involved all settings regardless of the type of health care facility or geographical location”; iii) “the study reported at least one outcome related to the environmental impact, resource use, or cost implications of green surgery in OTs”; and iv) “the study was published in English and related to human health care.” In addition, if studies were not categorised in the healthcare sector, were unrelated to surgery, or were published before 2013, they were excluded. Studies categorised as conference abstracts, posters, oral communication, or textbooks were excluded from the screening phase. Furthermore, studies from secondary research (i.e., literature reviews, comments, letters, and editorials), irrelevant articles, and duplicates were excluded.

### Search Strategy and Study Selection

**Figure 1:**
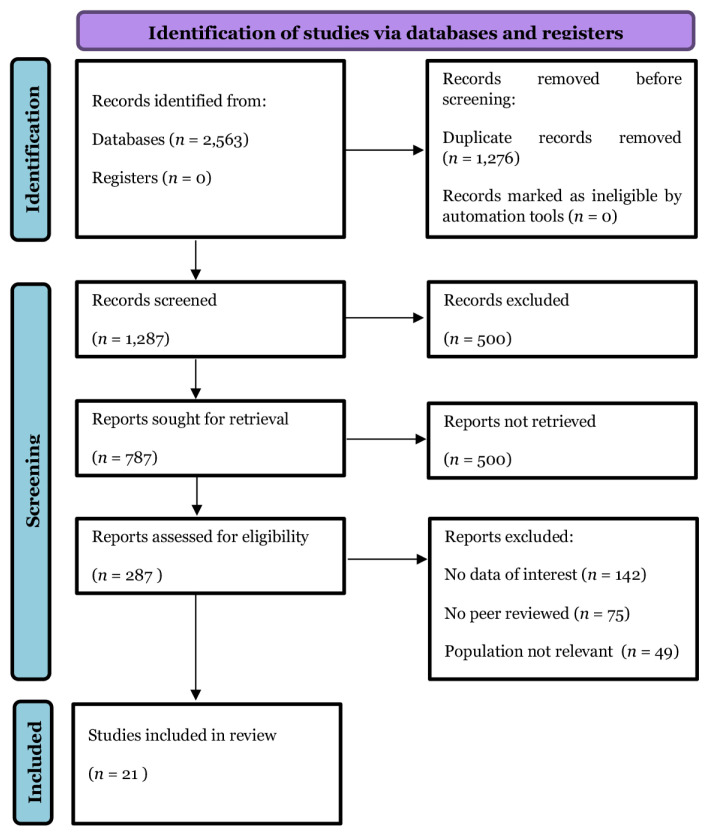
Study search and selection process summary using PRISMA flow diagram (2020 version). This flow diagram of the study search and selection process for the randomised controlled trials details the progression from the original PubMed, Central, MEDLINE, and other source research (*n* = 2,563) through duplication removal (*n* = 1,276), eligibility screening (*n* = 287) and finally to study inclusion (*n* = 21). *n* = number of studies.

A literature search was conducted across the following five databases: PubMed, ScienceDirect, Scopus, ProQuest, and Web of Science. A search strategy was designed to identify articles published between 2013 and the present. The keywords and Booleans used in the literature search were (“Green surgery” OR “sustainable surgery” OR “eco-friendly surgery” OR “low-carbon surgery”) AND (“healthcare system” OR “hospital sustainability” OR “medical sustainability”) AND (“carbon footprint reduction” OR “carbon emission reduction” OR “environmental impact” OR “greenhouse gas reduction”) AND (“operating theatres” OR “operating rooms” OR “surgical suites”) AND (“CPG Malaysia” OR “Malaysian clinical practice guidelines” OR “Malaysian healthcare”) AND (“recommendations” OR “implementation strategies” OR “best practices”). To limit the occurrence of undesirable articles, these keywords and Medical Subject Headings terms were searched in the “Title/Abstract” category. After collecting the studies from the database, the authors exported them to Excel software (Microsoft Corp, United States) for duplication removal and screening. Two independent review authors screened the titles and abstracts of the articles before scrutinising their full-text. Any disagreements between the two authors were resolved by discussion with a senior reviewer until consensus was reached. The excluded studies are described in the PRISMA flow diagram along with the reasons for exclusion (Figure 1).

### Study Outcomes

The primary outcomes of this guideline focus on reducing the carbon footprint of the OTs. As healthcare systems contribute significantly to global environmental pollution, OTs are a major source of hospital emissions owing to their high energy use and waste production. The goal was to evaluate the effectiveness of green surgical interventions, particularly those that focus on minimising waste and energy consumption, in reducing these emissions. The secondary outcome of this guideline was the financial, resource, and patient-related impact of implementing green practices in surgery beyond carbon footprint reduction. This is a crucial secondary outcome because initial investments in sustainable technologies often lead to significant long-term financial benefits.

Data regarding these outcomes were extracted from the included studies. Data on primary and secondary outcomes were systematically extracted to support evidence-based recommendations. For carbon footprint reduction (primary) outcomes, data were analysed according to trends across healthcare systems in countries that have adopted green surgical practices, such as the UK, the US, and Germany. Key interventions such as reusable instruments, biodegradable packaging, and waste segregation were assessed for their impact on energy use, waste production, and emissions. For financial and patient-related impact (secondary) outcomes, data were analysed using metrics, such as cost savings from energy-efficient technologies and assurance of sustainable practices, such as the use of reusable surgical instruments and linens without compromising patient care. These findings collectively strengthen the recommendations for integrating green practices into CPGs in Malaysia.

### Data Extraction and Collection

Two authors extracted the relevant data using structured and standardised forms. The extracted data included: i) study characteristics (author, year of publication, country of origin, and study design); ii) intervention characteristics (types of green surgery practices or technologies used); and iii) outcome data (environmental impact, resource use, cost implications, and patient outcomes).

### Quality and Risk of Bias Assessments

We followed predefined criteria to assess the Risk of Bias (RoB) of the included studies. Prior to analysis, the literature must undergo a quality assessment. The Cochrane’s RoB 2.0 tool was used to evaluate bias resulting from the randomisation process, intended intervention deviations, missing outcome data, outcome measurement, bias in choosing the reported result, and overall RoB (11). The researchers considered the articles based on “high risk,” “some concerns,” or “low risk” of bias using RoB Tool 2.0. The reviewers reconciled any disagreements until mutual agreement was achieved.

## Results

### Study Selection

The keyword “Green surgery” yielded 2,697 results, the combination of “Green surgery” and “healthcare system” produced 199 results, the combination of “Green surgery,” “healthcare system,” and “carbon footprint reduction” generated five results, and the combination of “Green surgery” and “operating theatres” resulted in 23 hits. A total of 2,563 papers were identified and 1,276 duplicate titles were excluded. A further 500 articles were eliminated; eventually, 787 articles met all the eligibility criteria. Of these, 500 articles were excluded owing to a lack of full-text articles, and 287 full articles were reviewed. Articles with no data of interest (*n* = 142), not peer-reviewed (*n* = 75), or irrelevant populations (*n* = 49) were excluded. After reviewing the selected articles, only 21 were deemed relevant and included in the final writing. A PRISMA flow diagram is shown in Figure 1.

## Discussions

### Green Surgical Practices

Emerging research highlights the need to minimise the use of disposable instruments and promote recycling in OTs (8). In the US, 25% of the solid waste generated from ORs is anaesthetic solid waste, of which 60% is recyclable (5). The UK is considered a pioneering country that has developed and implemented green surgery initiatives (9). For example, National Health Service (NHS) states that supply chains for medical and surgical supplies account for a large percentage of the carbon footprint (5). They emphasise the use of reusables over single-use instruments, which have been documented to decrease the carbon footprint of surgery by up to 50%–97% when disposable instruments are changed to reusable ones (3). In countries such as Germany, considerable attention has been paid to waste management in OTs, even extending it to the recycling of packaging materials. The results of the “Green Endoscopy Project in Würzburg” reduced CO_2_ emissions from waste disposal by 20.1% (10).

Formation of “Green Teams” in various departments within hospitals: These teams educate staff regarding waste reduction, energy conservation, and sustainable procurement (11). In a pilot study, development of a “Green Team” allowed for the appropriate separation of waste and induced behaviour changes that favoured high levels of environmental and financial savings with no compromise on patients or staff safety. The “Green Team” consists of many health professionals that were vital in the process of minimising biohazardous wastes by carefully collecting and separating in the ORs (4). The teams educate their staff on waste reduction, energy conservation, sustainable procurement, and integrating environmental sustainability into daily practices. After establishing environmental sustainability guidelines for the ICU and implementing intervention programmes, periodic progress evaluations should be conducted to assess the effectiveness of such programmes and their eventual effects on the ecological footprint of the ICU (7).

### Energy Consumption in OTs

OTs play a significant role in maintaining the sustainability of healthcare facilities because of the need for lighting, ventilation, air-conditioning, and medical equipment (1). It has been estimated that HVAC systems account for 90%–99% of OT energy consumption and are therefore a highly energy-intensive part of hospitals (5). Potential solutions include installing automatic lighting systems with occupancy sensors, which reduces energy usage by one-third per OR (12). Other effective measures include the installation of motion detectors in existing buildings, corridors, and staircases, and the construction of new endoscopy rooms (11). The installation of light-emitting diode (LED) lighting and renewable energy resources, including solar energy, is also a potentially effective way to reduce energy consumption (7). One study showed that changes in the HVAC system in a single hospital managed to decrease energy usage by 50%, further elucidating the potential for energy conservation programmes (13). In addition, placing solar panels in OT and hospitals would reduce fossil fuel consumption and power vital appliances and equipment (5).

### Waste Management and Recycling

OTs produce a large amount of medical waste, most of which involves single-use plastics. One study estimated that single-use plastic devices accounted for up to 23% of greenhouse gas emissions, mainly due to the production of polyvinyl chloride (PVC) (12). Environmentally friendly packaging and biodegradable replacements for plastic packaging are also under consideration to reduce environmental load (1). In fact, products made from bio-based materials, such as reusable medical devices, have significantly lower carbon footprints than their disposable counterparts (12). Biodegradable materials are increasingly being used to produce medical products that decompose naturally and help reduce waste (5). In addition, reusable equipment made of gowns and masks manufactured from sustainable materials is a highly effective waste-reducing strategy with a negligible carbon footprint compared with single-use alternatives (14). Vozzola et al. (15) conducted a life cycle comparison of gowns used for patients in isolation and reported that reusable gowns used 28% less energy, released 30% less greenhouse gas, and required 50% less water. Similar results were observed in cases of reusable surgical lines (11). Reusable surgical instruments and equipment, which are safe and feasible, have been found to substantially reduce the volume of OT waste substantially (1). Research conducted on laryngoscope blades, for example, has proven that reusable steel blades emit 25 times less greenhouse gas than their single-use plastic counterparts (12).

Biogenic coatings, such as chitosan extracted from shellfish, also represent new types of biomedical device coatings aimed at limiting the wastes produced while deposing them after applications (16). Metal and plastic recycling processes, including green manufacturing utilising renewable resources, also form part of this environmental sustainability in health care, and “reduce, reuse, recycle, and rethink” have been the point of talk together with the introduction of Lean Six Sigma (7).

Evidence supports the implementation of recycling systems and waste segregation protocols to reduce overall medical waste volume (7). Maximising recycling led to a 2% decrease in greenhouse gas emissions per case compared with the baseline. Reusing towels (2%), reusable linens (2%), reprocessing single-use devices (13%), and minimal instruments (64%) have resulted in a reduction in emissions per case (12). This reduces the demand for raw materials and minimises the carbon footprint of OTs (14).

### Green Leadership Development Through Training, Monitoring, and Sustainable Practices

Although there is some awareness of global warming, there is a lack of leadership and education among the surgical staff regarding this issue. This requires formal training programmes aimed at raising awareness and reducing the environmental impact (2). Harris et al. (17) conducted a survey between June and November 2020 and received responses from 130 surgeons and surgical trainees in the UK and Ireland. The results showed that 122 of 130 respondents (94%) were concerned about the risks associated with climate change. However, lack of leadership was the main barrier to progress in sustainability initiatives (17), cited by 70% (92 of 130 respondents). This could only be addressed by commencing the task of providing comprehensive training programmes in OT staff to gain insight into the global surgical knowledge base, and current efforts in surgical sustainability help provide a clear sense of these initiatives. Targeted OR interventions may include major carbon footprint reduction by reducing surgical waste, enhancing OR and perioperative processes to consume less energy, favouring reusable instruments whenever possible, and improving segregation at the source to enhance appropriate recycling and disposal of each type of waste (1).

Additionally, forming “Green Teams” by all the operational roles within the facility with each responsible for executing environmental initiatives can play a key role in fostering a culture of environmental awareness (7). In June 2021, Nicola Leone, Department of Surgical Sciences of the University of Turin, Italy and her multidisciplinary team launched the “O.R. Goes Green” project together with the Technical University Delft, and the Dutch OR Waste recycling consortium called “Green Cycle.” The “Green Team” was established, consisting of two surgeons, one anesthesiologist, one head nurse, and two hospital managers with sustainability expertise. The OR staff received regular information and training on waste management. Meetings were conducted to apprise all people about the new practices to be followed. This created a recycling programme in the OT, which cut the carbon footprint from biohazardous waste to a large extent while reducing the cost of hospitals (4).

The proposed 3Rs green team strategies support these green surgical practices to reduce the environmental impact of OTs, such as reducing the carbon footprint of surgical equipment through renewable energy resources and transitioning to reusable tools that significantly reduce water usage and substantially decrease pollution (7). Thus, life cycle assessments (LCA) can be implemented in addition to healthcare and product practices that enable data-driven decision-making by decreasing the environmental footprint of medical institutions (14). Moreover, other LCA studies clearly explain that reusable instruments have smaller ecological footprints (1). Following sustainable waste management practices, for instance, the rule of 5R’s: reduce, reuse, recycle, rethink, and research) has improved environmental impact reducibility (11).

### Carbon Footprint Reduction Strategies

Some countries have adopted a holistic approach to reducing the carbon footprint of hospitals. Australia, for example, has developed large-scale programmes such as the National Health Sustainability and Climate Unit and the National Health and Climate Strategy, reflecting a countrywide commitment to healthcare sustainability. These programmes foster energy efficiency and prudent use of resources in ICUs and other medical facilities, with promising results in terms of waste reduction (7). Key strategies include retrofitting hospitals with sustainable technologies, such as installing energy-efficient HVAC systems, LED lighting, and motion sensors, to optimise the preservation of energy (7). In Belgium, a position statement was proposed on 26 October 2023 by M. Aerts et al., in cooperation with the Vlaamse Vereniging voor Gastro-Enterologie (VVGE), the Société royale belge de Gastoentérologie (SRBGE), and the Belgian Society of Gastrointestinal Endoscopy (BSGIE), considering the change in renewable energy sources for endoscopy rooms, automatic lighting systems, and motion sensors for OTs along corridors and staircases as part of an eco-friendly hospital. The positive statement also indicated that the policymakers and governments of Belgium, along with their local gastroenterology and endoscopy societies, VVGE, SRBGE, and BSGIE, are bound to raise awareness and lobby for amendments in the reimbursement criteria. Therefore, it is essential to understand these issues be understood (16).

Standard practice, such as using alternative anaesthetic agents when clinically appropriate, in concert with the standard practice of reusing linen and gowns, effectively reduces carbon emissions (12). A study comparing the environmental impact of reusable operating room bed cover and lift sheets versus single-use bed covers, led by the team of Jenny H. Chang in Cleveland, Ohio, USA, further showed that the use of washable bed covers has a relatively lower environmental burden than single-use bed covers (14). For instance, in Germany, a project called the ‘Green Endoscopy Project Würzburg’, which ran from February to May, was led by Dorothea Henniger’s team and Thomas Lux. The project recorded a 10% reduction in the number of instruments used per procedure and approximately 20.5% of items that switched to alternative instruments, thus contributing to an 18.4% decrease in carbon emissions (10).

Based on some of the above-mentioned case studies and strategies, the following valuable insights are provided for a country such as Malaysia to encapsulate its CPG for environmental sustainability in healthcare practices to promote global sustainability.

### Review of Malaysia’s Existing Guidelines on Healthcare Sustainability

While Malaysia is moving to appreciate the concern for environmental sustainability, many formalised guidelines targeting carbon footprint reduction in healthcare facilities have been underdeveloped. While the country has made great strides in aligning its health goals with the 2030 Agenda for Sustainable Development, especially through Sustainable Development Goal (SDG) 3 (Good Health and Well-being) and other health-related SDGs, a more comprehensive approach is needed to address sustainability in high-impact areas such as OTs. Current environmental initiatives in healthcare mostly revolve around the problems of solid waste management, clean water and sanitation, and injury-all related to urbanisation, which has been gaining momentum. Over time, the nexus among urban poverty, environmental issues, and health has assumed a degree of relevance. Thus, these subjects occupied a major thrust in the SDG and Universal Health Coverage (UHC) goals and indicators. However, in general, actionable steps for carbon footprint reduction in OTs have not yet been institutionalised into detailed CPGs (18).

The health sector contributes greatly to global carbon emissions (2). ORs contribute greatly to health facilities’ environmental footprint due to the high utilisation of energy and consumables, along with the high volumes of waste generated therein (19). For that reason, Malaysia has to start developing a green surgery initiative whereby energy efficiency, along with other practical waste management approaches aimed at the OTs, contributes towards achieving a reduction in utility cost for reduced carbon emissions, pointing towards the health and well-being aims of healthcare. This may be achieved by enhancing HVAC systems to make them more energy-efficient, embracing proper management of waste disposal, revising preference cards to enhance the process of sorting waste, and shifting procurement towards ‘greener’ surgical consumables (1). Carbon calculator tools can also be used to effectively quantify carbon reduction strategies (10). What will further enhance the sustainability of Malaysia’s healthcare system, especially in high-impact areas such as OTs (1), are collaborative efforts from surgical societies with global initiatives and stronger policy enforcement.

### Identification of Gaps Where CPG Can Integrate Specific Actions for Reducing OTs’ Carbon Footprint

There are many serious lacunae in the existing environmental guidelines in Malaysia concerning theatre operations (18). The general guidelines advocate for efficient waste disposal but lack focus on OTs, where HVAC systems are one of the major consumers of energy (9, 18). Integrating strategies, such as optimising HVAC systems, using energy-efficient lighting, and exploring renewable energy sources, can significantly contribute to reducing carbon emissions (7). Inclusion of waste management under the broader scope of environmental health but failure to distinguish high-waste areas such as OTs (18). Sustainable practices in OTs, such as promoting reusable surgical instruments and implementing proper waste disposal and recycling systems, are missing from the current framework (1). Anaesthetic gases used in OTs, such as desflurane, contribute to the emission of greenhouse gases. In addition, it contributes more than isoflurane or sevoflurane. Lower-impact alternatives or encouraging Total Intravenous Anaesthesia (TIVA) may decrease the environmental burden (19).

Additionally, the development of the staff education tool and establishment of the “Green Teams” has been underemphasised (7). Multidisciplinary cooperation among surgeons, anesthesiologists, nursing staff, wardsmen, hospital administrators, and the local government is emphasised, which helps in the development and integration of protocols or policies that minimise the carbon footprint of OTs (13). Several studies further necessitate the need to review carbon footprints in the chains of diagnostic imaging, considering that some have failed to understand what emissions occur during the manufacturing process, installation, and decommissioning of these imaging devices, which should ideally have formed the core of developing strategies that ensure reduced emissions in healthcare (20). Radiology departments can find ways to reduce energy consumption by refining facility layouts, adopting remote work practices, implementing tracking equipment, and optimising data storage. Further research into the sustainability framework in diagnostic urology is still needed, questioning and improving existing practices (21). These gaps in the CPGs of Malaysia have the potential to significantly improve the environmental impact of OTs and align the healthcare system with global sustainability goals.

### Financial Impact

Although sustainable practices can involve higher initial expenses, they tend to yield strong long-term financial returns in the long run (12). By adopting energy-efficient technologies and processes, hospitals can experience significant cost savings by reducing energy consumption and waste management expenses. For instance, switching to LED lighting and energy-saving HVAC systems have been shown to reduce energy costs (7). Reusable equipment was initially more expensive but yielded significant financial savings over time. It yielded financial benefits only when the items in these packs were meticulously tracked and retained to avoid frequent replacement. Attempts to reduce and streamline resource usage yield financial benefits in the short- and long-term (12). Instead of selling surgical instruments individually, selling them as sets could reduce the costs incurred by hospitals and, at the same time, reduce carbon emissions from hospitals (12). Energy-efficient systems contribute to reducing operational costs, particularly in areas of high consumption, such as OTs (1). These strategies provide economic and environmental benefits.

### Environmental Impact

The potential reduction in carbon emissions from the adoption of sustainable practices in the OR is substantial (8). Replacing single-use items with biodegradable alternatives and reusable materials can significantly reduce hospital carbon emissions (12). For instance, LCA shows that reusable linens, when laundered and reused, have a far lower carbon footprint than disposable alternatives (14). Hospitals that have implemented renewable energy sources have significantly reduced their greenhouse gas emissions over time (12). The use of biodegradable materials and diversion of uncontaminated plastic waste for recycling help minimise the waste sent to landfills (9). It can be assumed that because single-use endoscopes are manufactured for a single use, they have a greater carbon footprint from manufacturing and transportation, which also produces more waste aside from that resulting from the procedure compared to natural resources (9). Accordingly, hospitals can reduce energy consumption by implementing renewable energy sources and green technologies (7). Moreover, green surgery can reduce environmental and air pollution (1). Modern anaesthetic gases are often under-recognised as sources of greenhouse gas emissions. Despite their relatively small contribution, they still have a notable effect on total annual emissions (22). A 2022 study by Griffin et al. demonstrated that transitioning from general anaesthesia to spinal anaesthesia in appropriate surgical procedures resulted in a decrease in annual carbon emissions (12).

### Key Recommendations

The sustainability framework that OTs in Malaysia may adopt must focus on waste reduction, energy efficiency, and eco-friendly medical practices. Guidelines on avoiding single-use items, changing to energy-efficient technologies, and promoting recycling can easily reduce the footprint of hospitals (18). First, waste reduction can be achieved by using reusable medical instruments, avoiding single-use items, and educating staff about the proper segregation of waste (1). Leaner surgical trays and the use of recyclable packaging for surgical supplies can significantly reduce the amount of waste generated (1). For example, In June 2021, the main OR of the Department of General Surgery of the University of Turin successfully reduced biohazardous waste by carefully separating clean waste from contaminated materials, resulting in decreased incineration and CO_2_ emissions (1). In another instance, carbon emissions were reduced by 40%–66% compared to single-use alternatives by reprocessing single-use devices and using reusable linens in hospitals (12).

Second, energy efficiency should be directed toward the adoption of energy-efficient lighting and ventilation systems, and the optimisation of equipment use (1). For example, John Radcliffe Hospital in Oxford, UK, was able to reduce energy consumption by 50% after modifying its HVAC system, thus demonstrating the potential for such interventions (2). Technologies such as heat-recovery systems and automatic lighting systems in OTs can contribute to substantial energy savings (1). In addition, the implementation of occupancy-based ventilation systems has demonstrated a one-third reduction in energy use, further supporting the use of smart energy management in OTs (2). In addition, conversion into LED lighting in OTs not only reduces energy consumption but also contributes to better comfort for caregivers (13). Finally, promoting the use of environmentally friendly medical practices, such as low-impact anaesthetic gases or intravenous anaesthesia, can further reduce the carbon footprint. For instance, one study demonstrated that a reduction in the use of the high-impact anaesthetic gas desflurane led to a ten-fold decrease in anaesthetic gas emissions, thus showing the potential for the use of low-impact alternatives (11). In addition, intravenous anaesthesia introduced in a few hospitals has also resulted in a massive reduction in the carbon footprint of surgeries (12). The promotion of minimally invasive surgeries that consume fewer resources is crucial for long-term sustainability (1). Evidence suggests that minimally invasive procedures, despite requiring more packaging and plastics, still have the potential to lower overall waste and emissions when optimised (5) (Table 1).

### Integration of Sustainable Practices into CPG Malaysia

Effective strategies must be developed to integrate sustainable practices into CPG for broader adoption in Malaysian hospitals. This includes embedding sustainability in clinical protocols and decision-making processes (16). For instance, a study conducted in three hospitals in the UK, Canada, and the USA showed that OTs were three to six times more energy-intensive than other areas of the hospital, and the most significant contributors were heating, ventilation, and air-conditioning (HVAC) requirements (2). The implementation of energy-efficient strategies in these areas is essential for reducing carbon emissions (2).

Potential barriers such as high upfront costs and resistance to change among healthcare professionals are significant challenges (6). Overcoming these issues can be achieved through strategies such as providing financial incentives for hospitals to adopt sustainable practices, securing government support, and promoting educational initiatives to foster a culture of environmental responsibility in healthcare. Furthermore, stakeholder involvement at all levels, from policy makers to grassroots health workers, is necessary for the successful implementation of sustainable practices (18) (Table 2).

### Global Comparisons

Case studies in other countries have served as a rich source of information on how other nations have successfully implemented health-based policies. A study in the UK, Canada, and USA-based surgical suites, for instance, showed that surgery is three to six times more energy-consuming than other departments of a hospital; this is primarily due to HVAC systems (2, 19). These countries have pursued various strategies to reduce energy consumption through HVAC setbacks to enhance sustainability while continuing to meet standards of care (2, 19).

The NHS in the UK, being a frontrunner in healthcare sustainability, promised to achieve net-zero emissions by 2045, and focused on ORs as a priority. Interventions are focused on reusing tools and using sustainable energy resources to change surgery from a high-carbon contributor to a greener, more responsible healthcare area (5). The Cleveland Clinic in the US, with its sustainability initiative, exemplifies a comprehensive approach with methods spanning perioperative care, such as energy-efficient lighting, reusable surgical materials, and strict waste categorisation. This initiative not only reduces greenhouse gases but also operational costs, proving that environmental and economic goals can coexist (5). In June 2021, the main operating room of the Department of General Surgery of the University of Turin implemented waste segregation programmes that led to a significant reduction in biohazardous waste and optimisation of waste management systems, demonstrating how environmental and financial savings can be achieved with sustainable waste management (4).

Countries with strong governance structures have been instrumental in coordinating efforts by the public and private sectors, NGOs, and civil society organisations to realise sustainable health goals. By integrating sustainability within health care, Malaysia has developed frameworks to reduce its environmental impact on the health sector. The Ministry of Health promotes green health care by enforcing responsible waste management and optimising energy consumption in hospital operations, aligning with the SDGs to ensure future health sustainability (18). Malaysia can also learn from countries that have streamlined UHC goals through inclusive governance models that emphasise intersectoral collaboration (9).

In addition, Malaysia can adopt similar mechanisms to enhance the frameworks for data collection, monitoring, and reporting to monitor gaps and progress in near real-time. Countries focusing on equity and the social determinants of health have managed to integrate these principles into their national health policies to realise significant improvements in health outcomes. Malaysia can replicate such models by ensuring that its health strategies not only focus on curative measures, but also preventive and holistic approaches to health (17).

### Global Challenges and Limitation to the Malaysian Context

There are several obstacles to the implementation of green surgery in Malaysia. Financial constraints are one of the main deterrents. In many situations, sustainable surgical equipment requires substantial upfront investments. When hospitals make supply chain decisions, they frequently concentrate on immediate costs, comparing the prices of disposable products to the upfront costs of reusable products. However, this study revealed the critical need to account for the lasting impact on the environment (14). Additionally, a lack of awareness among healthcare providers and the public regarding the environmental impact of healthcare operations contributes to the slow adoption of green initiatives (1). Limited awareness of the environmental effects of anaesthetic options is the main obstacle to adopting low-carbon practices (19).

Numerous challenges lie in translating the recycling, reuse, and reprocessing programmes of single-use devices into practice at a local level, including economic barriers, lack of physical space, and adherence to policies regarding infection prevention. There is a current trend in most medical specialties towards single-use products; life cycle analysis of different surgical options has to be performed for more detailed research in these areas, which must also consider patient safety concerns over cost-effectiveness (5). Other challenges include overcoming resistance to change, gaining stakeholder support, maintaining programme sustainability, and considering people’s tendency to fall back into their previous habits (7).

## Conclusions

OTs are among the largest contributors to the ecological footprint of healthcare systems because of their high energy usage, anaesthetic gases, and the waste generated by them. However, evidence-based strategies include the use of energy-efficient HVAC and different choices of anaesthesia (e.g., TIVA), and reusable surgical instruments that can be used to reduce the carbon footprint of OTs. Coupled with the above practices, integrating green surgery into Malaysia’s CPG will lead the healthcare industry to take a leap toward national sustainability. The introduction of green surgery in Malaysian hospitals would ensure that any initiative for sustainability aligns with the aim of future-proofing businesses in a world that keeps changing at a break-neck speed. Training programmes should be provided to health professionals to shorten the knowledge gap and provide insights into the application of sustainable practices in day-to-day operating activities.

## Figures and Tables

**Table 1:** Key recommendations on green surgery

Recommendations	Description(s)
Waste reduction (1)	Implement reusable medical instruments, minimise single-use items, and educate staff on efficient waste segregation. Use leaner surgical trays and recyclable packaging to reduce waste (1). Example: In June 2021, the University of Turin’s General Surgery Department reduced biohazardous waste by separating clean waste from contaminated materials, decreasing incineration and CO_2_ emissions (4). Reusable linens and reprocessed devices can cut carbon emissions by 40%–66% (12).
Energy efficiency (1)	Focus on energy-efficient lighting, ventilation, and optimised equipment use. Radcliffe Hospital (Oxford, UK) cut energy use by 50% with HVAC adjustments (2). Technologies like heat-recovery systems, automatic lighting, and occupancy-based ventilation reduce energy use (1). LED lighting lowers energy consumption and improves comfort (13).
Eco-friendly medical practices (11)	Transition to low-impact anaesthetic gases (e.g., reduce desflurane usage for a ten-fold decrease in gas emissions) or intravenous anaesthesia, both of which significantly lower the carbon footprint of surgeries (12). Minimally invasive surgeries, despite requiring more packaging, can reduce overall waste and emissions (1).

**Table 2:** Integration of sustainable practices into CPG Malaysia

Recommendations	Description(s)
Embed sustainability in clinical protocols (16)	Develop strategies to incorporate sustainable practices into CPG for widespread adoption across Malaysian hospitals. This involves integrating sustainability into clinical protocols and decision-making processes (16).
Reduce energy usage in OTs (2)	OTs are highly energy-intensive, especially in HVAC, which are major contributors to carbon emissions. Implement energy-efficient practices in these areas (2).
Waste management initiatives (4)	Example from University of Turin (June 2021): Waste segregation programme in the main operating room significantly reduced biohazardous waste, showing potential for environmental and economic benefits (4).
Address barriers (6)	Common barriers include high upfront costs and resistance from healthcare professionals. Overcome these by providing financial incentives, securing government support, and promoting educational initiatives to build a culture of environmental responsibility (6).
Stakeholder engagement (18)	Engage stakeholders at all levels, including policymakers and frontline healthcare workers, to ensure successful implementation of sustainable practices in healthcare (18).

## List of Abbreviations

CO_2_: carbon dioxide
CPG: clinical practice guideline
HVAC: heating, ventilation and air-conditioning
LCA: life cycle assessment
LED: light-emitting diode
NHS: National Health Service
OR: operating room
OT: operating theatre
PVC: polyvinyl chloride
RoB: Risk of Bias
SDG: Sustainable Development Goal
TIVA: Total Intravenous Anaesthesia
UHC: Universal Health Coverage

## References

[1] Johnson SM, Marconi S, Sanchez-Casalongue M, Francis N, Huo B, Alseidi A (2024). Sustainability in surgical practice: a collaborative call toward environmental sustainability in operating rooms. Surg Endosc.

[2] Chan KS, Lo HY, Shelat VG (2023). Carbon footprints in minimally invasive surgery: good patient outcomes, but costly for the environment. World J Gastrointest Surg.

[3] Robinson PN, Surendran K, Lim SJ, Robinson M (2023). The carbon footprint of surgical operations: a systematic review update. Ann R Coll Surg Engl.

[4] Leone N, Scozzari G, Olandese F, Horeman T, Passera R, Arezzo A (2024). “O.R. Goes Green”: a first step toward reducing our carbon footprint in the operating room and hospital. Updates Surg.

[5] Asfaw SH, Galway U, Hata T, Moyle J, Gordon IO (2021). Surgery, anesthesia, and pathology: a practical primer on greening the delivery of surgical care. J Clim Chang Health.

[6] Cannon J, Tailor H, Douglas C (2024). Carbon footprint of tonsillectomy. Surgeon.

[7] Masud FN, Sasangohar F, Ratnani I, Fatima S, Hernandez MA, Riley T (2024). Past, present, and future of sustainable intensive care: narrative review and a large hospital system experience. Crit Care.

[8] National Institute for Health and Care Research Global Health Research Unit on Global Surgery (2023). Reducing the environmental impact of surgery on a global scale: systematic review and coprioritization with healthcare workers in 132 countries. Br J Surg.

[9] Sebastian S, Dhar A, Baddeley R, Donnelly L, Haddock R, Arasaradnam R (2023). Green endoscopy: British Society of Gastroenterology (BSG), Joint Accreditation Group (JAG) and Centre for Sustainable Health (CSH) joint consensus on practical measures for environmental sustainability in endoscopy. Gut.

[10] Henniger D, Lux T, Windsheimer M, Brand M, Weich A, Kudlich T (2024). Reducing scope 3 carbon emissions in gastrointestinal endoscopy: results of the prospective study of the ‘Green Endoscopy Project Würzburg’. Gut.

[11] Beloeil H, Albaladejo P (2021). Initiatives to broaden safety concerns in anaesthetic practice: the green operating room. Best Pract Res Clin Anaesthesiol.

[12] Lam K, Gadi N, Acharya A, Beatty JW, Darzi A, Purkayastha S (2023). Interventions for sustainable surgery: a systematic review. Int J Surg.

[13] de’Angelis N, Conso C, Bianchi G, Barría Rodríguez AG, Marchegiani F, Carra MC (2024). Systematic review of carbon footprint of surgical procedures. J Visc Surg.

[14] Chang JH, Woo KP, Silva de Souza Lima Cano N, Bilec MM, Camhi M, Melnyk AI (2024). Does reusable mean green? Comparison of the environmental impact of reusable operating room bed covers and lift sheets versus single-use. Surgeon.

[15] Vozzola E, Overcash M, Griffing E (2018). Environmental considerations in the selection of isolation gowns: a life cycle assessment of reusable and disposable alternatives. Am J Infect Control.

[16] Aerts M, Reynaert H, Roelandt P, Caenepeel P, Dewint P, Lemmers A (2024). Position statement on how can we can implement the Greendeal in our gastrointestinal and gastrointestinal endoscopy department in Belgium. Acta Gastroenterol Belg.

[17] Harris H, Bhutta MF, Rizan C (2021). A survey of UK and Irish surgeons’ attitudes, behaviours and barriers to change for environmental sustainability. Ann R Coll Surg Engl.

[18] Ministry of Health Malaysia (2020). Health in the Sustainable Development Goals and Universal Health Coverage: progress report for Malaysia 2016–2019 and seminar proceedings. [Internet].

[19] MacNeill AJ, Lillywhite R, Brown CJ (2017). The impact of surgery on global climate: a carbon footprinting study of operating theatres in three health systems. Lancet Planet Health.

[20] Woolen SA, Kim CJ, Hernandez AM, Becker A, Martin AJ, Kuoy E (2023). Radiology environmental impact: what is known and how can we improve?. Acad Radiol.

[21] Woernle A, Moore CM, Allen C, Giganti F (2024). Footprints in the scan: reducing the carbon footprint of diagnostic tools in urology. Curr Opin Urol.

[22] Vanni G, Materazzo M, Pellicciaro M, Marino D, Buonomo OC (2023). From Patient Reported Outcome Measure (PROM) to Environment Related Outcome Measure (EROM): towards “Green Breast Surgery”. In Vivo.

